# Establishment of Colorectal Cancer Organoids in Microfluidic-Based System

**DOI:** 10.3390/mi12050497

**Published:** 2021-04-28

**Authors:** Diana Pinho, Denis Santos, Ana Vila, Sandra Carvalho

**Affiliations:** 1 International Iberian Nanotechnology Laboratory, Department of Nanoelectronics Engineering, 4715-330 Braga, Portugal; denis.santos@inl.int; 2 International Iberian Nanotechnology Laboratory, IP Exploitation and Knowledge Transfer, 4715-330 Braga, Portugal; Ana.Vila@inl.int

**Keywords:** microfluidics, patient-derived organoids, colorectal cancer, 3D model, drug screening

## Abstract

Colorectal cancer is the second leading cause of cancer death worldwide. Significant advances in the molecular mechanisms underlying colorectal cancer have been made; however, the clinical approval of new drugs faces many challenges. Drug discovery is a lengthy process causing a rapid increase in global health care costs. Patient-derived tumour organoids are considered preclinical models with the potential for preclinical drug screening, prediction of patient outcomes, and guiding optimized therapy strategies at an individual level. Combining microfluidic technology with 3D tumour organoid models to recapitulate tumour organization and in vivo functions led to the development of an appropriate preclinical tumour model, organoid-on-a-chip, paving the way for personalized cancer medicine. Herein, a low-cost microfluidic device suitable for culturing and expanding organoids, OrganoidChip, was developed. Patient-derived colorectal cancer organoids were cultured within OrganoidChip, and their viability and proliferative activity increased significantly. No significant differences were verified in the organoids’ response to 5-fluorouracil (5-FU) treatment on-chip and on-plate. However, the culture within the OrganoidChip led to a significant increase in colorectal cancer organoid-forming efficiency and overall size compared with conventional culture on a 24-well plate. Interestingly, early-stage and late-stage organoids were predominantly observed on-plate and within the OrganoidChip, respectively. The OrganoidChip thus has the potential to generate in vivo-like organotypic structures for disease modelling and drug screening applications.

## 1. Introduction

Colorectal cancer is the second leading cause of cancer death worldwide, and because of population ageing, the global burden is expected to grow to 2.5 million new cases in 2035 [1,2,3]. Although significant advances in the molecular mechanisms underlying colorectal cancer have been made, the clinical approval of new drugs still faces many challenges [4]. Cancer drug development is characterized by high failure rates in clinical trials, mainly because the preclinical models used in the drug development pipeline do not provide adequate information about drug efficacy or toxicity [5]. Molecular and cellular tumour heterogeneity between and within individual patients represent a landscape and a barrier for effective patient treatment [6,7,8]. Thus, predictive preclinical cancer models able to precisely replicate the human tumour biology and allow personalized anti-cancer therapy are urgently needed [9,10]. Orthotopic mouse models are considered to be poor human cancer models as they lack the features of the native tissue and do not recreate the human tumour microenvironment [11]. Moreover, conventional 2D cell cultures do not provide information about the complex interactions between the cancer cells, associated stromal components, and the physicochemical microenvironment. Lastly, more complex 3D in vitro models, such as transwell-based cell cultures and spheroids, do not reproduce the complexity observed in the 3D tissue architecture of living organs, and do not retain the mechanical cues that contribute to the tumour behaviour [12]. Microfluidic cell culture technology has emerged as a promising tool in cancer research as an alternative to animal and traditional cell culture models [13,14]. It is a low-cost technology that handles fluids at a nanoscale, enabling small quantities of samples and providing highly sensitive and high-throughput screening [15,16]. A variety of fabrication methods, aside from soft lithography, vinyl cutters (xurography), laser cutting, 3D printing, and micromilling, among others, are available [17,18]. Microfluidics-based systems have contributed to decreasing manufacturing time and costs by using cheaper materials and tools, allowing for new and advantageous physical behaviour, functionality, and qualities in microfluidic devices. Modelling cancer cell behaviour within the microfluidic device of tumour-on-chip, is highly physiological; enables co-cultures of different cell types; and offer precise control over physical, mechanical, and biochemical properties on the model. However, tumour-on-chip relies on pre-differentiated cells, often cell lines, and cannot emulate the histological and cellular complexity of the tumour and the surrounding microenvironment [19].

Organoid models have been synergistically combined with microfluidic technology [20,21]. Organoids are 3D culture models that recapitulate the organ architecture and in vivo counterparts’ functional features [22]. The generation of organoids derived from primary and metastatic patient tumours are reported. These patient-derived organoids are distinct from other cancer models in that they conserve the architecture, (epi)genetic, and phenotypic alterations of the original tumour [23,24]. More importantly, patient-derived organoids have been reported to successfully predict a therapy response in cancer patients and facilitate therapeutic decision-making [23,25]. Therefore, establishing patient-derived tumour organoids-on-a-chip may serve as more versatile and predictive preclinical models applicable to cancer drug discovery and personalized therapies. Micro-engineered organoid systems allow for precise control of the space and environment where an organoid is growing. Flow conditions, nutrient supply, shear stress, input–output, and organoid geometry are easily controlled. Furthermore, the complex cellular cross-talk of the native organ can be modelled by organoid-on-chip technology, and the inter-organoid variability is drastically decreased [20].

In this study, a microfluidic device was designed and fabricated through a milling process so as to culture and expand patient-derived colorectal cancer organoids on-chip (OrganoidChip). A culture medium was continuously injected into the distribution channels to provide the proper conditions for organoid growth. Viability assays of organoids cultured within the OrganoidChip were performed and compared with those cultured on conventional well-plates. Our experiments showed a significant increase in organoid viability and proliferation within OrganoidChip, as well as similar sensitivity responses of colorectal cancer organoids to 5-FU on-chip and on-plate. These observations demonstrate the potential of OrganoidChip to provide precise control of drug distribution, similar sensitivity, and improved growth culture conditions of colorectal cancer organoids. Distinct morphology features of colorectal cancer organoids were observed. The culture within OrganoidChip led to a significant increase in colorectal cancer organoid-forming efficiency and organoid size. More importantly, late-stage colorectal cancer organoids were established within OrganoidChip, highlighting the potential for this micro-engineered organoid device to generate in vivo-like organotypic structures for disease modelling and drug screening applications.

## 2. Materials and Methods

### 2.1. Design and Fabrication of a Microfluidic Platform for Organoid Culture

The molds for the microfluidic device used in this study were fabricated using a high-speed milling machine (FlexiCam Viper, FlexiCam, Eibelstadt, Germany). Micromilling is an alternative, low-cost, non-lithographic top-down technique and a fast method to address some significant microfabrication challenges and allow rapid prototyping. Using rotating cutting tools to remove or cut micro- or milli-scale features in several types of materials (acrylic, aluminium, among others), it is possible to generate molds or directly fabricate microfluidic channels. Recently, manufacturers have produced milling tools smaller than 100 microns, which have promoted micro-milling machines’ ability to fabricate microfluidic devices [18,26].

The device design was created using AutoCAD software (Autodesk AutoCAD 2018, Autodesk, San Rafael, CA, USA) and consists of a bottom layer containing four round wells with 6 mm in diameter and 2.5 mm of depth for cell seeding, and a top layer displaying one inlet channel distributed in four small channels of 1.5 mm × 1 mm (width × depth) and one outlet (2 mm of diameter). The top layer is crucial for the culture medium supply.

The microfluidic devices were fabricated in polydimethylsiloxane (PDMS; Ellsworth Adhesives Iberica, Spain), which was prepared as a two-part system with a mix ratio of 10:1 (*w*/*w*) or 20:1 (*w*/*w*) base/curing agent (top and bottom layers, respectively), poured over the molds, degassed, and cured for 1.5 h at 65 °C. The different PDMS ratios were used for the bottom and top layers to generate slight and reversible bonding when both were poured in contact. Following that, the PDMS was unmolded, and the inlet and outlet were made using a puncher of 1.5 mm. Both layers and tubes were sterilized with 70% ethanol (*v*/*v*) and were then exposed for 30 min to UV light. Afterwards, the bottom layer (20:1 PDMS ratio) was placed on a glass slide, and the top layer (10:1 PDMS ratio) was carefully aligned and placed over the bottom layer. Thus, the layers stuck to each other, creating contact bonding. Tubes were inserted into the inlet and outlet. After organoids seeding, the channels were filled with a culture medium. The microfluidic device was connected to a syringe pump (NE-1000, New Era Pump Systems, Farmingdale, NY, USA) with a working flow rate of 10 µL/h for a continuous medium supply.

### 2.2. Organoids Culture

Colorectal cancer organoid line Iso-50 was supplied by Cellesce, Ltd., (Cardiff, Wales, UK) and was cultured according to their instructions [27]. These tumour organoids were isolated by Cellesce, Ltd., from surgically-resected colorectal cancer material of patients, as previously described by Sato et al. [24]. The organoid pellet was resuspended in medium 3+, composed by Advanced DMEM/F12 supplemented with 1% GlutaMAX, 1% HEPES buffer solution, and 1% Penicillin/Streptomycin (all Life Technologies, Cramlington, UK). Afterwards, organoids were plated in Matrigel Matrix Basement Membrane Growth Factor Reduced (Corning, NY, USA) in 24-well plates and a microfluidic device (30 µL of blob). Following Matrigel polymerization, the organoids were overlaid with 500 µL of “complete” medium composed of medium 3+ supplemented with 1× B27 supplement, 1× N2 supplement (all from Invitrogen), and 1 mm N-acetyl-L-cysteine (Sigma, Darmstadt, Germany). To culture the organoids on-chip, the microfluidic device’s bottom layer (previously placed on a glass slide) was first pre-warmed at 37 °C. Iso-50 organoids were then plated in the Matrigel matrix in each microfluidic device’s well (30 µL of blob). The bottom layer was placed at 37 °C in an atmosphere of 5% CO_2_ to promote Matrigel polymerization. The microfluidic device was assembled with a top layer and tubes, as previously described, and carefully filled with the appropriate culture medium for the organoids’ growth. The device was connected to a syringe pump and the flow rate condition was defined as 10 µL/h. For the on-chip control, Iso-50 organoids were cultured within the microfluidic device without a continuous flow of a culture medium.

All of the cultures were maintained in humidified incubators at 37 °C in 5% CO_2_, and were monitored daily under a phase-contrast microscope (Nikon Eclipse TS 100, Tokyo, Japan). The culture medium was changed every 2–3 days, and organoids were passaged 1:4 every week.

### 2.3. Viability Assay

Iso-50 organoids were seeded at a density of 200 structures per well of 24-well plates or microfluidic device (in 30 µL of blob per well), and were cultured until day 8. The viability of the organoids was determined using the CellTiter-Glo 3D Cell Viability Assay (Promega, Madison, WI, USA), as follows: organoids were mixed with 100 μL of CellTiter-Glo^®^ 3D reagent, shaken for 5 min, and incubated for 30 min at room temperature. Luminescence was measured using a Microplate Reader (Synergy H1, Biotek, Winooski, VT, USA). Viability was monitored at days 0, 2, 4, 6, and 8.

### 2.4. Seeding Density Optimization by ATP Assay

The Iso-50 organoids in the culture were gently dissociated to the near single-cell population using TrypLE (Gibco, Life Technologies, Renfrew, Scotland, UK) before resuspension within a growth-factor reduced Matrigel. The individual cells were counted using a Trypan blue exclusion test (Gibco, Life Technologies, UK) and were seeded at a range of densities (from 100 to 1000 cells/µL of Matrigel dome) into a 24-well plate or microfluidic device (30 µL blob per well). The ATP measurement of the CellTiter-Glo 3D Cell Viability Assay (Promega, Madison, WI, USA) was then performed to evaluate the organoid proliferative ability according to the manufacturer’s instructions.

### 2.5. Organoid-Forming Efficiency Assays

The organoid-forming efficiency was determined by quantification of the organoid numbers (organoid-colony forming efficiency) and size. The total number of organoid structures per well was manually counted six days after seeding using a phase-contrast microscope at 4× magnification. The organoid area was measured by encircling the periphery of each organoid using ImageJ software (NIMH, Bethesda, Rockville, MD, USA). The organoid size was quantified at day 6 of culture by measuring the longest axis.

### 2.6. Treatment of Iso-50 Organoids with 5-Fluorouracil

**Figure 1:**
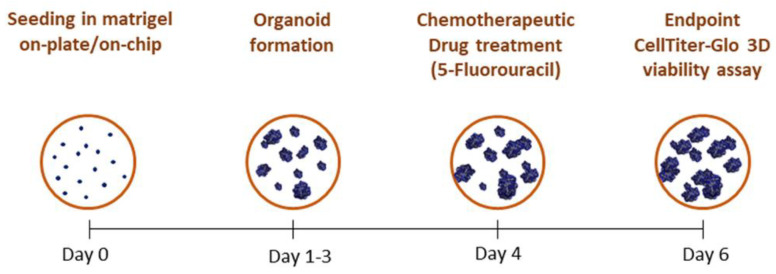
Schematic of the organoid culture timeline.

Colorectal cancer Iso-50 organoids were treated with chemotherapeutic drug 5-fluorouracil (5-FU) at different concentrations. Briefly, Iso-50 organoids were seeded in a 24-well plate and within the OrganoidChip. Complete media supplemented with 1, 10, and 100 nM 5-FU or 0.1% of dimethyl sulfoxide (DMSO) were replaced onto the organoids on day 4 of culture. DMSO was used as a vehicle for 5-FU. The viability of the organoids was measured after 48 h of treatment (Figure 1).

### 2.7. Statistical Analysis

Statistical analyses were performed using the Graph Pad program (GraphPad Software, Inc., La Jolla, CA, USA). Student’s tests were used to calculate significance with a 95% confidence level (* *p* ≤ 0.05; ** *p* ≤ 0.01; *** *p* ≤ 0.001).

## 3. Results

### 3.1. Fabrication of the OrganoidChip Microfluidic Device

The design considerations of the two layers were performed to have the possibility for manual seeding of the organoid structures. In this way, no cell damage and higher control in the seeding procedure could occur. There is also the possibility of downstream testing assays after the culture and maturation under continuous flow conditions.

The layer molds were fabricated in a poly(methyl methacrylate) (PMMA) material with single-flute carbide end-mill tools of 2 mm for the larger steps and 1 mm for the small features of the design and to smooth the surface. The depth of the bottom and top layer was 2.5 and 1 mm, respectively.

**Figure 2:**
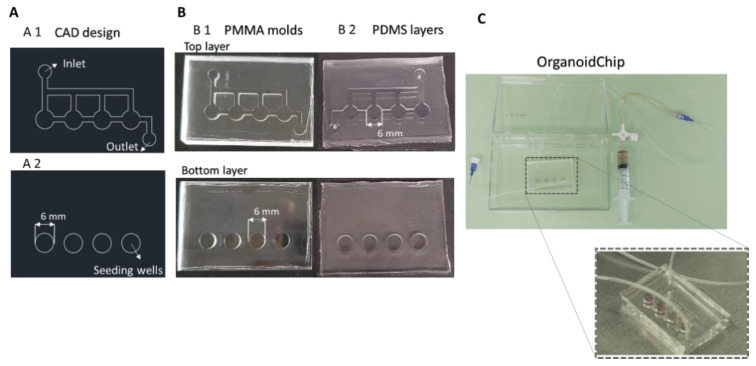
Design and fabrication of the organoid on-chip device. (**A**) AutoCAD design of the OrganoidChip (**A1**) top and (**A2**) bottom layers. The top layer includes one inlet channel distributed in four small channels and one outlet. The bottom layer consists of four round wells of 6 mm in diameter for organoids’ seeding and growing. (**B**) OrganoidChip poly(methyl methacrylate) (PMMA) layers mold fabricated in (**B1**) the milling machine and the (**B2**) respective polydimethylsiloxane (PDMS) layers. (**C**) OrganoidChip setup.

In Figure 2A, it is possible to observe the design of the manufactured PMMA molds. The PMMA material proportionated a clear and smooth surface in the PDMS device, essential for the microscope visualizations of 3D structures (Figure 2B). The inlet and outlet in the top layer had a diameter of 2 mm, and the channels for the medium supply had a width of 1.5 mm. The top layer was fabricated with 10:1 PDMS. The bottom layer had 6 mm round wells with a depth of 2.5 mm, and was fabricated with 20:1 PDMS. The PDMS thickness of the bottom and top layer was 4 mm and 2.5 mm, respectively. Thus, the OrganoidChip had a total thickness of 6.5 mm.

The bonding of both layers was performed by aligning the top layer over the bottom layer. An off-ratio bonding technique occurred (i.e., two layers partially cured with different base-to-crosslinker ratios are brought into contact creating a bonding interface) [28]. This technique enables layer adjustments and a contact bonding strong enough to support the working flow rate of 10 µL/h, giving the possibility of layer detachments and further applications. A flow rate of 10 µL/h has been used in several tumoroid platforms and reported to generate internal shear stress supported by the Matrigel blob [29,30]. Note that the established flow rate proportionates for 48 h had the same amount of medium added in the on-plate for the comparison tests (500 µL per well). With the inlet connected to a syringe pump via tubing and the outlet connected to a reservoir, the OrganoidChip setup was set, as seen in Figure 2C.

### 3.2. Establishment of Colorectal Cancer Organoid Line within OrganoidChip

**Figure 3:**
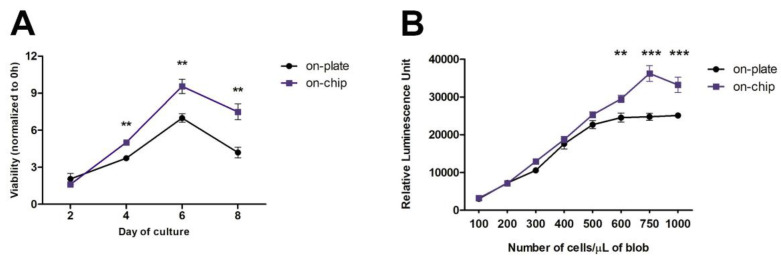
Optimization of Iso-50 organoids culture on-chip. (**A**) Viability of cultured organoids within OrganoidChip (on-chip) and on a traditional plate (on-plate). Viability was monitored at days 0, 2, 4, 6, and 8 using a CellTiter-Glo 3D Cell Viability Assay. The results are described as mean ± standard deviation (SD) of three independent experiments. The viability of the organoids at days 2, 4, 6, and 8 is expressed as the fold increase, compared with the viability at day 0, taken as 1 (Student’s *t*-test: ** *p* ≤ 0.01; *** *p* ≤ 0.001). (**B**) ATP measurement for proliferation in Iso-50 organoids after six days of culture at different cell seeding densities. Results are described as mean ± SD of three replicated wells (one independent experiment) for each cell density (Student’s *t*-test: ** *p* ≤ 0.01; *** *p* ≤ 0.001).

Having designed and fabricated the microfluidic device, the OrganoidChip (Figure 2), we explored its feasibility for culturing organoids. Proper development and maturation of organoids implies a continuous feed of fresh nutrients and waste removal, as they expand in size when they are not vascularized [31,32]. In this study, to evaluate the organoids’ growth, colorectal cancer Iso-50 organoids were seeded in Matrigel on-chip and on-plate (around 200 structures/well), and their viability was assessed over eight days of culture. As shown in Figure 3A, the organoids’ viability increased, as expected in the first days of culture. However, a significant decrease in viability was observed after six days of culture, both on-plate and on-chip. This result suggests that the exponential growth of organoids occurs within the first six days of culture. However, comparing the viability of the organoids in the different culture conditions, we observed that the viability of Iso-50 was significantly higher on-chip after day 2.

Moreover, the significantly decreased viability of the organoids observed from day 6 was more pronounced in the on-plate. These observations indicate that OrganoidChip provides a continuous infusion of nutrients and growth factors, and removes metabolic waste, which are crucial for developing organoids within the microfluidic device. As the organoids viability increases significantly until six days of culture, the endpoint at day six was pointed out all subsequent culture experiments (Figure 1).

We further evaluated the influence of the cell seeding density on the organoids’ proliferative ability. Single-cell suspensions were prepared from Iso-50 organoids. The cells were seeded at a range of cell densities within a traditional 24-well plate and microfluidic device, and their proliferation was assessed on day 6 of the culture by measuring the ATP released from the proliferative cells composed of organoids. Regarding the organoids cultured on-plate, an increase in the relative luminescence signal was observed with the ascent of the initial cell seeding on-plate (Figure 3B). Beyond 600 cells/µL of blob, a plateau was reached, indicating a slowdown in the organoid proliferation activity, possibly due to restriction to the nutrient and growth factor access and the limited physical space. Interestingly, such a threshold was observed at a seeding density of 750 cells/µL of blob on-chip, highlighting that on-chip and under continuous flow conditions, it is possible to have more proliferation. The continuous flow proportionates the constant and renews the culture medium.

### 3.3. Culture within OrganoidChip Promotes Colorectal Cancer Organoid-Forming Efficiency

**Figure 4:**
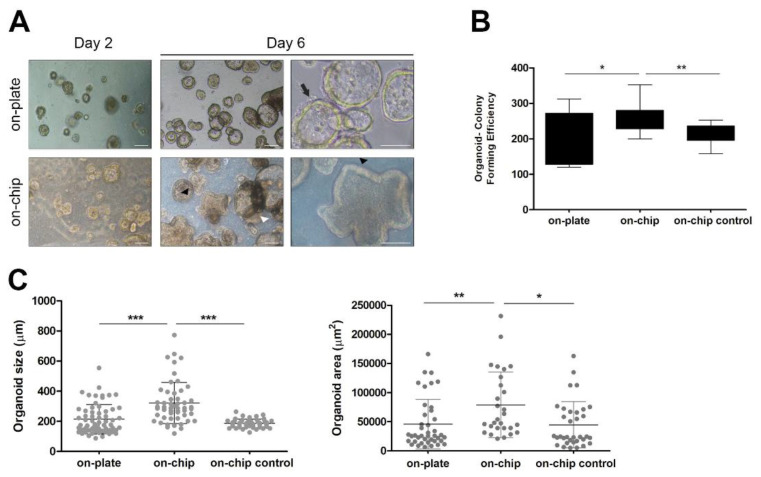
Organoid-forming efficiency within OrganoidChip. (**A**) Representative images of organoids morphology at days 2 and 6 of culture. On day 6, Iso-50 organoids cultured on-plate exhibited a cystic-like morphology with a visible lumen. In contrast, the organoids cultured on-chip are multi-layered with crypt-like projections or display a thick epithelial cell layer containing glandular structures. Scale bar: 100 µm. (**B**) Quantification of organoid colony-forming efficiency at day 6 of culture. The results are reported as the mean ± SD of three independent experiments. (**C**) Analysis of organoid size and area at day 6 of culture. The results are described as mean ± SD of a total of 50 organoids in three independent experiments subjected to the analysis (Student’s *t*-test: * *p* ≤ 0.05; ** *p* ≤ 0.01; *** *p* ≤ 0.001).

The colorectal cancer organoids were characterized to display the morphological and histological features in common with the tumour from which they were derived [27]. The morphological characteristics of the Iso-50 organoids were observed on days 2 and 6 of the culture on-plate versus on-chip (Figure 4A). On day 2 of the culture, colorectal organoid formation was still occurring, as the not late-stage morphology typical of colorectal cancer organoids was not verified. Small and round organoids were observed both on-plate and within OrganoidChip. On day 6 of the culture, distinct morphology features were observed. Iso-50 organoids cultured on-plate were predominantly mono-cellular structures with a cystic-like shape, exhibiting a visible lumen without projections (arrow). In contrast, structures displaying a thick epithelial cell layer containing glandular structures within the organoid (white arrowhead) or multi-layered organoids with crypt-like projections (black arrowhead) were observed when cultured within OrganoidChip. Because of these different morphological features of Iso-50 organoids cultured on-chip versus on-plate, the organoid-forming efficiency was evaluated. The organoid-forming efficiency was determined by quantifying the organoid numbers (organoid-colony forming efficiency) and size (are and longest axis). The organoid-colony forming efficiency was significantly higher on-chip than on-plate and on-chip control without continuous flow (Figure 4B). In addition, Iso-50 on-chip organoids were significantly higher in diameter and area than for the culture on-plate and on-chip control (Figure 4C). These results highlight the potential of OrganoidChip to provide more physiologically relevant cell culture conditions than the static cell culture, thus contributing to the efficient establishment of cancer organoids.

### 3.4. On-Chip Organoids Drug Sensitivity Testing

**Figure 5:**
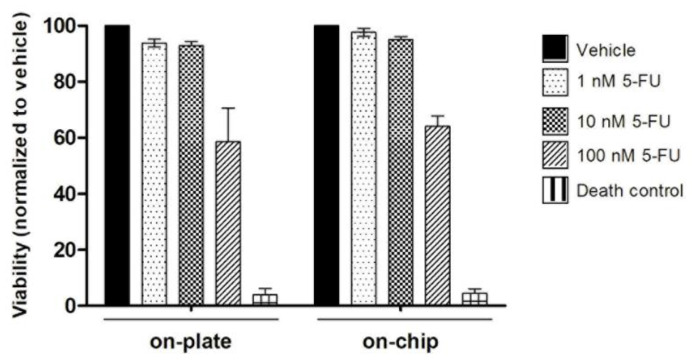
Analysis of organoid viability after treatment with 5-fluorouracil (5-FU). On day 4 of culture, the organoids were treated with 5-FU or 0.1% dimethyl sulfoxide (DMSO; control), and their viability was assessed on day 6. For the dead control, 30% of DMSO was used. The results are described as the mean ± SD of two wells per condition of two independent experiments. No significant differences were observed (Student’s *t*-test: * *p* ≤ 0.05; ** *p* ≤ 0.01; *** *p* ≤ 0.001).

To assess the organoids’ sensitivity cultured within OrganoidChip to chemotherapeutic drugs, Iso-50 organoids were treated with an increasing dose of 5-fluorouracil (5-FU). 5-FU remains the first-line of treatment for colorectal cancer. Following treatment with 5-FU for 48 h, the organoid viability was determined. According to Figure 5, no significant differences in sensitivity to 5-FU treatment were observed in the colorectal cancer organoids cultured within OrganoidChip and traditional plates. These results suggest the stability and accuracy of colorectal cancer organoids cultured within OrganoidChip as a drug screening model.

## 4. Discussion

In the present study, a microfluidic device—OrganoidChip—was fabricated using a milling technique to enable the culture of patient-derived colorectal cancer organoids on-chip. The milling fabrication technique was employed to create the master molds of the microchannels and the main body of the OrganoidChip. In the last decades, milling and micromilling have demonstrated a great potential for lab-on-a-chip and organ-on-a-chip applications, primarily because of their wide selection of working materials, versatile applications, and rapid prototyping, compared with other fabrication procedures [33]. The most common milling application is the fabrication of organ-on-chip platform holders [34] or the direct use of the milled parts combined with other methodologies, such as photolithography and 3D printing, to connect all of the device modules, as Behroodi et al. demonstrated [35]. Another rapid, reliable, and cost-effective microfabrication of microfluidic systems with biomedical applications and lab-on-a-chip devices is the CO_2_ laser [chen, wu]. Chen et al. successfully elaborated a versatile protocol of CO_2_ laser drilling for the rapid prototyping of various microstructures with different substrate materials to showcase the wide usability range for the proposed method [36]. Wu et al. developed a micro U-well platform with an optimally arranged microwell array using a rapid and straightforward CO_2_ laser microfabrication method. This platform has demonstrated their feasibility for developing in vitro 3D multicellular tumour spheroids as a tumour-mimicking model, and as an effective tool for discovering the therapeutic drug screening for cancer treatment [37]. Besides the advantages of CO_2_ laser patterning, the milling fabrication for this application is demonstrated to be equally efficient, but cheaper and easier to integrate into the developed device’s manufacturing. In this work, the layer molds were fabricated in acrylic to obtain a higher durability, and PDMS replica molding was performed. In this way, a transparent device with an adequate refractive index for microscope visualizations and image acquisition and gas permeability was obtained. Another important application for the developed system is the possibility of downstream studies. The organoids could be recovered intact, as the layers are not bonded permanently, and no holder strategies were needed. Thus, the milling fabrication simplified the manufacturing process of the OrganoidChip: low- cost, high precision, and accuracy in the molds’ manufacturing, as well as being user-friendly and applicable for downstream studies.

We further investigated the potential for OrganoidChip as a reliable preclinical cancer model. For this, patient-derived colorectal cancer organoids were cultured within the OrganoidChip. The establishment of patient-derived organoids is crucial for preclinical cancer research and personalized medicine, as they retain patients’ genetic and epigenetic aspects. Currently, various patient-derived tumour organoids have been generated. Using microfluidic technology, most reported organoid-on-chips rely on organoids derived from human induced pluripotent stem cells [38,39,40]. Tumour organoids derived from patient tissues with lung, mesothelioma, breast, and pancreatic cancer have been generated and cultured within microfluidic devices [41,42,43,44]. In our study, colorectal cancer organoids derived from patient tissue established by Cellesce and reported in pilot studies with reproducible data, maintaining the counterpart tissue’s phenotype and genotype, were used [27]. Their viability and proliferation, when cultured within OrganoidChip, was evaluated. Our results revealed significantly increased viability and proliferation rates of colorectal cancer organoids on-chip, compared with traditionally cultured on-plate. Consistent with our observations, previous reports have shown a significant improvement in cell growth and survival within microfluidic devices that can minimize shear stress. In fact, microfluidic technology allows dynamic cell cultures in microperfusion systems to deliver continuous nutrient supplies for long term cell culture [45,46].

Patient-derived organoids preserve the structural architecture of primary tumours [22]. Our results show that colorectal cancer organoids cultured within OrganoidChip exhibit a significant increase in organoid size and organoid colony-forming efficiency, as well as a late-stage morphology, compared with organoids cultured on-plate. Previous reports have shown that as tumour organoids grow in size in conventional culture plates, the diffusion-dependent nutrient and oxygen supplies and waste removal become less efficient. Consequently, organoid viability decreases, and dead cells accumulate in the core region of organoids and undergo fragmentation and necrosis [46,47]. In contrast, microfluidic devices enable the integration of access channels for nutrient supply within organoids, as well as waste removal, mimicking the permeation provided by blood capillaries in vivo [47]. In our study, the OrganoidChip provides a control overflow, shear stress, and biochemical gradient. Nutrients, metabolic, and oxygen are delivered to organoids via laminar flow, reducing the necrotic core’s size and increasing their viability, organoid colony-forming efficiency, and overall size.

The nutrient and oxygen supplies and the removal of waste provided by microfluidic technology also influences organoid maturity. Interestingly, the significant morphological differences observed between colorectal cancer organoids cultured within OrganoidChip and on-plate were closely related to the organoid’s organization stage. The colorectal cancer organoids cultured within OrganoidChip exhibited a late-stage morphology, characterized by a crypt and villi-morphology, thick epithelial cell layer, and oriented and specialized epithelial cells. In contrast, we observed early-stage organoids on-plate as monocellular structures with a cystic-like shape. In fact, the organoid formation process followed a pattern of proliferation, differentiation, cell sorting, lineage commitment, and morphogenesis, resulting in a 3D organoid structure [48,49]. Organoid formation is usually guided by culturing cells in a medium containing soluble factors that promote or inhibit specific signaling pathways. Thus, the culture is directed towards the formation of late-stage organoids, which includes specialized cell types that give rise to organotypic structures and functions [50].

The potential for microfluidic devices for the culture and expansion of patient-derived organoids for personalized drug screening is of the utmost importance. In this study, similar sensitivity responses of colorectal cancer organoids to chemotherapeutic drug 5-FU were observed within OrganoidChip and on-plate. These results highlight the potential for OrganoidChip to provide precise control of drug distribution, sensitivity to chemotherapeutic drugs, and improved growth culture conditions of colorectal cancer organoids. Microfluidic platforms able to culture patient-derived pancreatic and lung tumour organoids and to perform drug sensitivity tests directly on devices have been developed [41,44]. These studies observed significant differences in the responses of individual patient-based organoids to drug treatments.

Taken together, we fabricated a low-cost microfluidic device suitable for the maintenance and expansion of patient-derived colorectal cancer organoids, applicable in downstream studies. The organoids’ morphological and proliferation features were improved when cultured within this microfluidic device. More importantly, a high-fidelity response to drug treatment was observed compared with the organoid culture in traditional plates. However, device optimizations are needed in order to obtain a higher versatility for the design for other applications. Several outlets can be added; seeding chambers area and flow rates can also be adapted and optimized considering the final application. The individual response of patient-based organoids to 5-FU drug treatment is also necessary. Additionally, further studies, including the genetic profiling of individual patient-based organoids on-chip, are warranted to validate OrganoidChip as a reliable preclinical cancer model.

Overall, these results open new avenues for evaluating phenotypic drug susceptibility tests and disease modelling, as well as for the development of an organoid-on-chip preclinical cancer model with the potential for personalized medicine.
